# Microinverter Thermal Performance in the Real-World: Measurements and Modeling

**DOI:** 10.1371/journal.pone.0131279

**Published:** 2015-07-06

**Authors:** Mohammad Akram Hossain, Yifan Xu, Timothy J. Peshek, Liang Ji, Alexis R. Abramson, Roger H. French

**Affiliations:** 1 Department of Mechanical and Aerospace Engineering, Case Western Reserve University, Cleveland, Ohio, United States of America; 2 Solar Durability and Lifetime Extension (SDLE) Center, Case Western Reserve University, Cleveland, Ohio, United States of America; 3 Center for Statistical Research, Computing and Collaboration, Department of Epidemiology and Biostatistics, Case Western Reserve University, Cleveland, Ohio, United States of America; 4 Underwriters Laboratories, Northbrook, Illinois, United States of America; 5 Department of Material Science and Engineering, Case Western Reserve University, Cleveland, Ohio, United States of America; 6 Department of Macromolecular Science and Engineering, Case Western Reserve University, Cleveland, Ohio, United States of America; 7 Department of Physics, Case Western Reserve University, Cleveland, Ohio, United States of America; University of California Berkeley, UNITED STATES

## Abstract

Real-world performance, durability and reliability of microinverters are critical concerns for microinverter-equipped photovoltaic systems. We conducted a data-driven study of the thermal performance of 24 new microinverters (Enphase M215) connected to 8 different brands of PV modules on dual-axis trackers at the Solar Durability and Lifetime Extension (SDLE) SunFarm at Case Western Reserve University, based on minute by minute power and thermal data from the microinverters and PV modules along with insolation and environmental data from July through October 2013. The analysis shows the strengths of the associations of microinverter temperature with ambient temperature, PV module temperature, irradiance and AC power of the PV systems. The importance of the covariates are rank ordered. A multiple regression model was developed and tested based on stable solar noon-time data, which gives both an overall function that predicts the temperature of microinverters under typical local conditions, and coefficients adjustments reecting refined prediction of the microinverter temperature connected to the 8 brands of PV modules in the study. The model allows for prediction of internal temperature for the Enphase M215 given similar climatic condition and can be expanded to predict microinverter temperature in fixed-rack and roof-top PV systems. This study is foundational in that similar models built on later stage data in the life of a device could reveal potential influencing factors in performance degradation.

## Introduction

In the past decade, renewable energy has seen remarkable growth, especially in the development of photovoltaic (PV) energy systems. The growth in PV is primarily influenced by the declining cost of PV modules [1]. The International Energy Agency (IEA) predicts that by 2050, the cumulative global PV capacity will reach around 3000 GW and meet 11% of the demand for global electricity [2]. Studies in [3, 4] show that 20%-30% PV integration to the grid can be accommodated through: 1. Use of demand response and system balance, 2. Increased flexibility of dispatchable generation, 3. Advanced forecasting in fast market operations, and 4. Greater system interconnections and faster scheduling. For example, Plug in hybrid electric vehicles (PHEVs) and electric vehicles (EVs) are potential sources of dispatchable loads [5–8]. Integrated energy storage or battery storage can store the excess PV power in low demand periods and reduce the problem of variability. An essential component of the integration of PV system to grid is the inverter that converts the DC output of the PV module to utility frequency AC. String inverters and microinverters are two widely used types of inverter systems used in a PV system. In a string inverter system, a number of PV modules, electrically in series connection with each other, are connected together and the cumulative total DC power generated by the connected PV modules is supplied to the string inverter. On the other hand, a microinverter is designed to connect with one PV module where the AC power output from all of the inverters is in parallel, i.e. there are no series connections in this system. One advantage of microinverters is that maximum power point tracking (MPPT) is performed on a per module basis and contains no single point of failure of the whole PV system [9]. Per module MPPT eliminates the effects of module mismatch and reduces the effect of shading, which are very common in residential PV systems [10].

It is critical for microinverter designers and developers to fully understand the environment in which their products exist. Microinverters are usually installed outdoors underneath the PV modules, and they have to endure a wide variety of climate conditions, including temperate (moderate), tropical (warm damp equable), and desert (extremely warm dry), for example [11, 12]. These different climate conditions can induce different degradation mechanism in the microinverters than observed for string inverters. Modern commercial microinverter manufacturers go through accelerated lifetime test (ALT) based on IEC61215 [13] to determine the reliability of the microinverter [14]. Real-world operation is a unique combination of multiple stressors. Indoor accelerated test can introduce single or several stressors however they do not simulate the precise combination of multiple stressors to mimic real-world operation.

Metal-oxide-semiconductor field-effect transistors (MOSFETs), capacitors, inductors, diodes, transformers, and circuit boards are considered as the critical to lifetime performance (CLP) components for string inverters and microinverters [15, 16] and have various potential failure modes. Typically electrolytic capacitors are used in microinverters and the operating lifetime of these capacitors are limited by the operating environment temperature. The aqueous component of electrolytes evaporates at higher operating temperature, and increase the equivalent series resistance (*R*_ESR_) of the capacitor [17, 18]. As a consequence of this ESR increase, more heat accumulates inside the capacitors, accelerates the evaporation rate, reduces the capacitance and eventually leads to capacitor failure with positive feedback. Although electrolytics receive much attention, MOSFETs are considered the most failure prone component in the inverter system with thermal stress being the dominant stressor [19, 20]. Thermal stress can develop in the die package due to rapid heat build up in the die as observed in the case of an insulated-gate bipolar transistor (IGBT) during power cycle [21, 22]. Typically inductors are the hottest component in an inverter [16]. Thermal stress and cycling, especially in the presence of a plastic resin pottant, that many microinverters contain, may crack the sintered ferrite material comprising the inductors and dramatically alter the inductance or saturation field characteristics. Therefore, predicting thermal behavior or the temperature of the microinverter in the real-world operation can provide design insights, and more accurate lifetime modeling and help to assess the system reliability. Furthermore, for accurate modeling of reliability for an outdoor technology there must be appropriate models of the thermal performance in the field given real-world exposure. All components are dependent upon temperature, and in particular most design parameters and conventional lifetime estimation models are closely dependent upon an estimate of field and use temperatures. However, conventional lifetime estimation models do not take into account device degradation or the scientific mechanism of degradation.

In real-world operation, thermal stresses on microinverters can be derived from several heat and energy sources. The typical DC to AC power conversion efficiency of microinverter is approximately 96% [23], and the remaining 4% lost energy is converted into heat among the components. Another important source of thermal stress for microinverters can be the radiant heat arising from the PV module backsheet temperature. PV modules warm up by absorbing the solar insolation, and radiates the heat energy from the warm backsheet of the PV module. The microinverter, located beneath the PV modules, receives the PV module backsheet radiated heat and eventually aids in the development of increased thermal stress in the microinverter. Ambient temperature, wind speed and irradiance are some other important factors behind the development of thermal stress that we studied to develop the predictive model for microinverter temperature.

There are very limited studies conducted for microinverters as a component of full PV systems, or on temperature prediction of the microinverters. However, the PV community has been trying to develop PV module temperature predictive models, and these models can be studied as references for microinverter temperature prediction. King *et al*. [24] and Faiman [25] have proposed theoretical and empirical models for predicting PV module temperature as a function of weather data: irradiance, ambient temperature and wind speed, respectively. Kurtz *et al*. [26] used an equivalent PV module temperature to study thermal degradation of the PV module. Koehl *et al*. [27] used principal component analysis to identify the main influencing factors behind PV module temperature, and developed a statistical model and a simple analytical model to predict PV module temperature from weather data. However, these models did not consider the influence of radiation and natural convection. There are also few studies that considered heat conduction between PV cells [28], front sheet and back sheet, and convection, both natural and forced [29, 30]. Several predictive models for PV module temperature employ the energy balance equation in conjunction with empirical equations [31]. The effect of PV module inclination, wind velocity and direction on PV module temperature has been studied and a simulated model was developed to predict PV module temperature [32]. However, this predictive model did not include the effects of clouds or rapidly changing environmental conditions where the steady state thermal model is not valid.

Our infrastructure is designed to handle data acquisition, validation and cleaning of real-world time series datastreams that allows for insights to be gained and data-driven predictive models to be generated. We apply a degradation science methodology to gain insights into the thermal performance of microinverters using data science [33], or an agnostic approach that relies on exploratory data analysis and statistical practices to further scientific knowledge, past examples include our work on PV modules’ degradation pathways under damp heat [34], acrylic degradation [35], and transparent conducting oxides [36]. We apply these methods and develop a parametric, time-independent regression model of microinverter temperature given the rank-ordered predictors.

The remainder of this paper is structured as follows. In the Experimental Setup and Metrology Platform section, the outdoor real-world test setup and data collection methodology is described. The Data analysis section describes the data cleaning and validation techniques, exploratory data analysis techniques and the modeling methods and principles followed to develop the predictive model of microinverter temperature. The impacts of different predictors on microinverter temperature, thermal behavior of PV modules and microinverter in different times of the day, the accuracy and limitations of the predictive model are discussed in the Discussion section. Finally, conclusions and future work directions are presented in the Conclusion section.

## Experimental Setup and Metrology Platform

In this study, 24 Enphase M215 microinverters connected to 8 different brands of polycrystalline PV modules on dual-axis trackers were analyzed from July through October 2013 in SDLE SunFarm (latitude 41.50°, longitude -81.64°) on the campus of Case Western Reserve University in Cleveland, Ohio. Three PV modules from each brand are connected to the microinverters. The rated output power of this microinverter is 215 W, and peak output power is 225 W. The 24 microinverters are distributed in 3 different tracker sites of the SunFarm: tracker 6, 12, and 14. The baseline power of different brands of PV modules are listed in Table 1. Baseline power was measured by the SPIRE4600 solar simulator [37]. In Table 1, the letters “K”, “L”, “O”, “P”, “Q”, “R”, “S” and “T” represent the different PV module brand names and t# corresponds to the site and tracker location, for example: K.t6 represents PV module brand K at tracker 6.

**Table 1:** Baseline DC power of different brands of PV modules.

Module Brand	Baseline power (W)	Standard deviation (W)
K.t6	225.19	2.59
L.t6	231.02	4.12
O.t12	241.14	1.65
P.t12	231.75	0.95
Q.t12	212.86	4.98
R.t14	231.38	1.53
S.t14	230.95	1.03
T.t14	232.20	2.47

**Fig 1:**
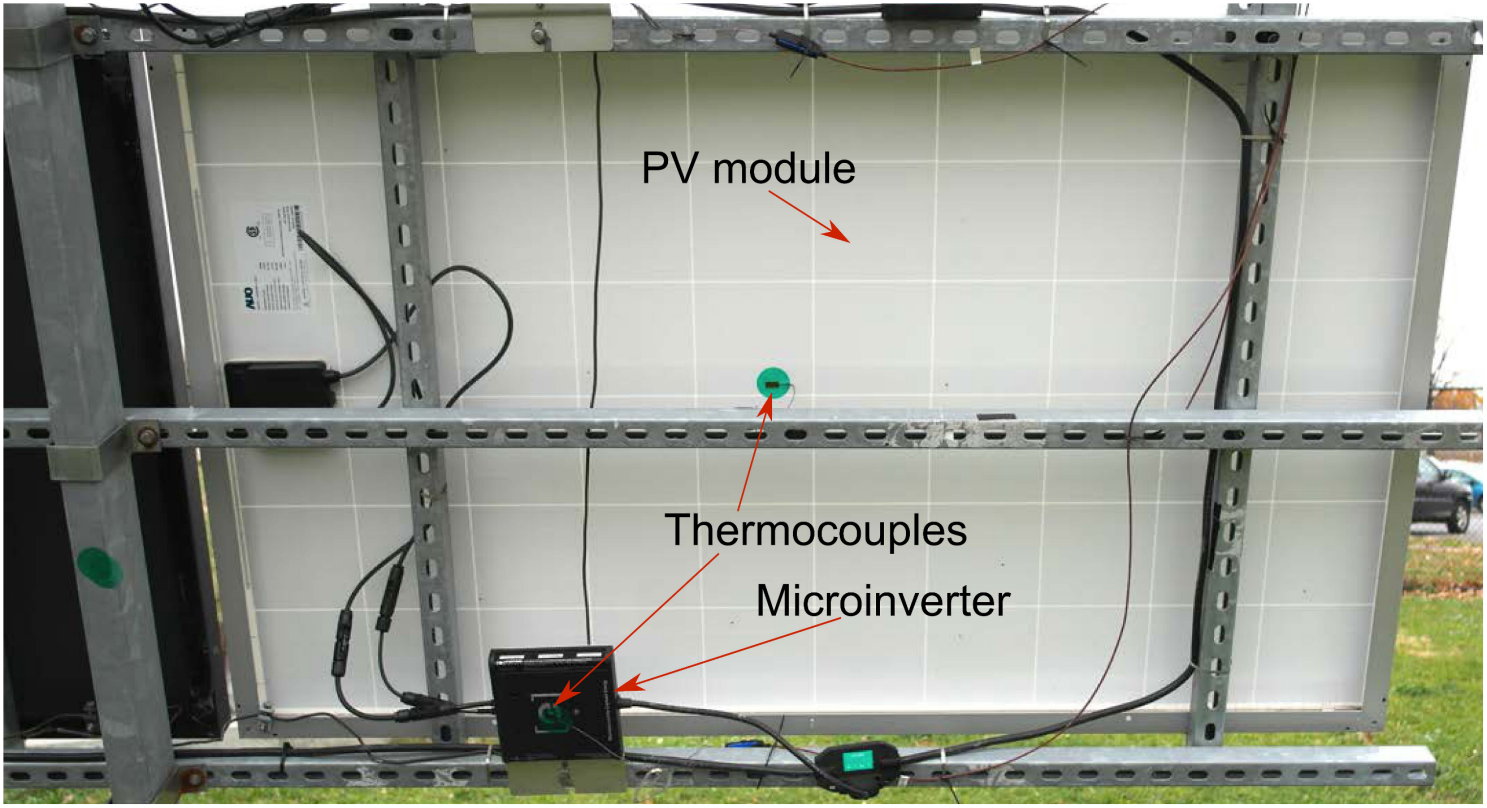
PV module and microinverter setup in a dual-axis tracker in SDLE SunFarm. PV module and microinverter setup in a dual-axis tracker with thermocouple attached to the hottest points of the PV module and the microinverter.

A Kipp & Zonen CMP11 pyranometer was used to measure insolation data and a Vaisala WXT520 weather transmitter collected the environmental data: ambient temperature, wind speed, wind direction, relative humidity, and rain intensity. T-type thermocouples (CO1-T) from Omega Engineering Inc. were used to measure the PV module backsheet temperature and the microinverter peak temperature. The thermocouples were attached to the middle of the backsheet of the PV modules and the hottest point on the microinverters (Fig 1) as determined by infrared (IR) thermography collected at the maximum rated input power using a FLIR T300 camera [38]. The pyranometer, weather transmitter and the thermocouples reports the data to the Campbell CR1000 data loggers [39] and multiplexers [40] at a one minute time interval. The data loggers store all the collected data in the central database every two hours. An Enphase Envoy device maintains power line communication with the Enphase microinverters to collect the inverter telemetry: DC voltage and current, and AC power, frequency, and microinverter internal temperature and reports those data to the Enphase enlighten website every 5 minutes. The power data was automatically acquired from the Enphase enlighten website using the Java Selenium web driver package and stored in SDLE Center’s local file-store. Later, all these data: power, insolation, temperature and climate, were ingested into SDLE center’s informatics and analytics infrastructure, known as Energy CRADLE [41]. Data visualization and analytics in this manuscript were generated using ‘R’ open-source software [42, 43].

## Data Analysis

A time lag exists between data collected from disparate data sources, due to asynchronicity of internal clocks and varying sensitivity to daylight savings time. The time lags of different data sources were determined using a cross correlation function (ccf) and it was found that the Enphase Enlighten system reported power data that was lagging approximately 1 hour 6 minutes behind the environmental, temperature, and insolation data reported by the Campbell systems. The power data were then slewed to match the environmental, temperature, and insolation data.

Thermocouples were attached using purpose specific special polyester adhesive tape on the backsheet of PV modules and microinverters (Fig 1) to report the backsheet temperature data [44]. The thermocouple reported temperature data were plotted together with ambient temperature and Enphase reported microinverter internal temperature. If any thermocouple reported temperature trends with the ambient temperature closely, even under high insolation when the inverter was under full load, then it was assumed that the thermocouple delaminated from the backsheet and is reporting ambient temperature. This assumption was then confirmed by field investigation and those data where the external thermocouple matched ambient temperature at high insolation were excluded from further analysis [45].

### Exploratory Data Analysis

Exploratory Data Analysis (EDA) [46] was conducted to detect outliers, check preliminary assumptions, find patterns and trends, summarize the characteristics of data sets, and suggest appropriate statistical models. Pairwise scatter plots for multivariate graphical EDA were generated that provide us the graphical overview of the relations between all pairs of variables, and correlation coefficients were also calculated. Note that the correlation coefficient measures the *linear* correlation between two variables. It ranges from -1 to 1, where -1 indicates strong inverse correlation, 0 means no correlation, and 1 indicates strong positive correlation.

**Fig 2:**
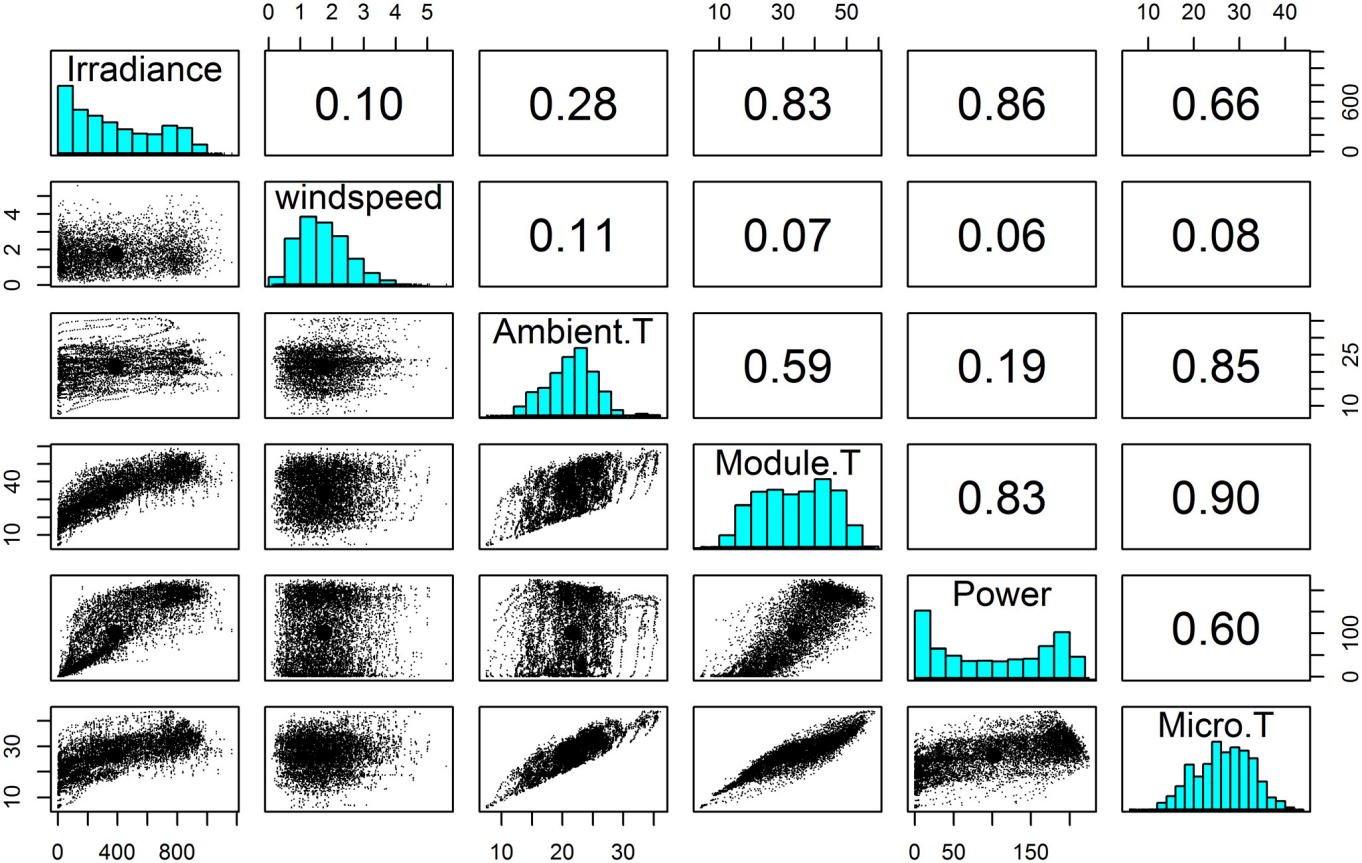
Pairwise scatter plot matrix, histogram and correlation coefficients of all related variables for the microinverters connected with different brands of PV module. Pairwise scatter plots are in lower triangle boxes, histograms are in the diagonal boxes, and upper triangle boxes give the correlation coefficients between variables. Brand, Ambient. T, Wind speed, Module. T, Power and Micro. T denote PV module brand, ambient temperature, 5 point moving average wind speed, PV module temperature, AC power output and microinverter temperature respectively.

Fig 2 shows the pairwise scatter plots (lower half), histograms and corresponding correlation coefficients (upper half) among irradiance, wind speed, ambient temperature, module temperature, AC power and microinverter temperature, for the cleaned data set [47, 48]. The ambient temperature and PV module temperature are strongly correlated with the microinverter temperature. Additionally, AC power, and irradiance are also moderately correlated with the microinverter temperature. However, the wind speed shows little correlation with any other variables measured in this study.

Table 2 summarizes the brand-dependent maximum temperature of the PV modules and the connected microinverters where Module.T_*M*_, Micro.T_*M*_, ΔModule.T_*M*_, and ΔMicro.T_*M*_ denote the maximum PV module temperature, the maximum microinverter temperature, the maximum temperature difference between PV module temperature and ambient temperature, and the maximum temperature difference between microinverter temperature and ambient temperature respectively. For this study, the average ambient temperature is 21.54°C with standard error (SE) 0.02°C (maximum observed ambient temperature is 36.09°C, minimum temperature is 7.5°C and median temperature is 1.63°C), and average wind speed is 1.73 *m*/*s* (maximum, minimum and median wind speed are 5.592 *m*/*s*, 0.108 *m*/*s* and 1.63 *m*/*s* respectively). Maximum PV module temperature are observed in Q.t12 and T.t14 respectively, and maximum microinverter temperature are observed in T.t14 and Q.t12 microinverters respectively.

**Table 2:** Maximum PV module temperature, PV module temperature difference with ambient temperature, maximum microinverter and microinverter temperature difference with ambient temperature during study period.

Brand	Module.T_*M*_	ΔModule.T_*M*_	Micro.T_*M*_	ΔMicro.T_*M*_
	(°C)	(°C)	(°C)	(°C)
P.t12	58.47	34.23	43.98	15.79
O.t12	58.09	32.36	43.46	13.01
Q.t12	59.20	33.51	44.17	12.99
R.t14	55.00	32.66	42.30	13.62
S.t14	56.39	32.20	41.74	12.65
T.t14	59.16	35.76	44.28	13.52
K.t6	55.75	30.57	42.60	13.73
L.t6	58.72	32.23	43.39	12.90

**Fig 3:**
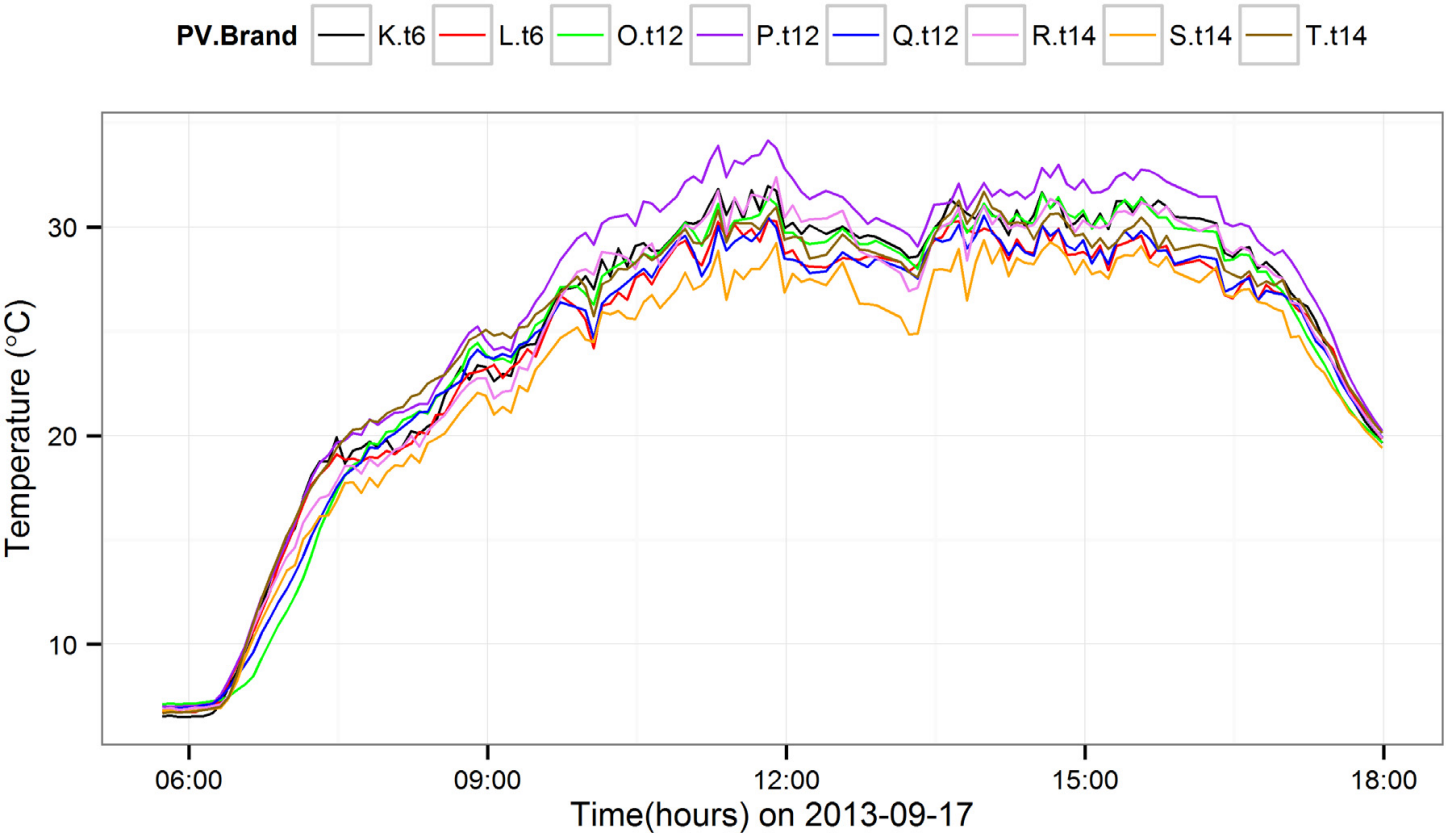
Comparison of average microinverters temperature on a typical sunny day. Comparison of average microinverters temperature connected to 8 different brands of PV module on a sunny day (date: 2013-09-17).

Fig 3 is a plot of daily thermal variation of microinverter as a function of PV module brand on a typical sunny day (2013-09-17). During the low irradiance morning hours, the temperature variance between the microinverters connected to the different PV module brands is very small. The temperature variance is comparatively higher during noontime. This also indicates a duality of thermal performance behavior according to the time of day (morning and noontime).

**Fig 4:**
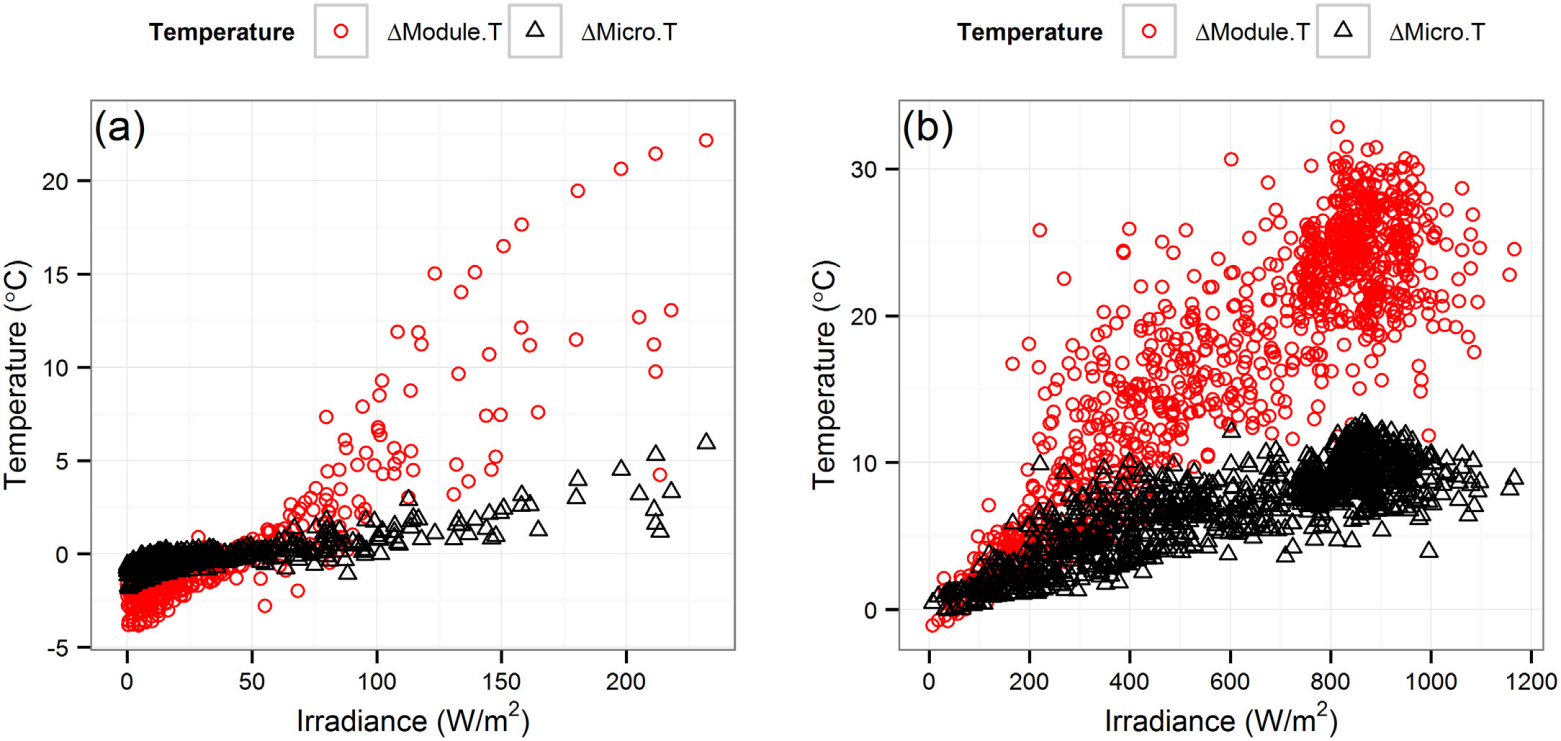
Variation in microinverter temperature and PV module temperature with irradiance in (a) the morning, and (b) noon time. Variation in microinverter temperature and PV module temperature with irradiance for Q.t12 PV microinverter and PV modules in the morning and noon time.

According to the distinct thermal characteristics of microinverters in different time ranges, we segregate two subsample data sets, dubbed “morning” and “noontime”, to isolate and observe the thermal attributes under relative low and high irradiance conditions. Morning time is defined as local solar time (LST) [49] from 05:00 to 06:30, and the noontime dataset is defined between LST from 10:00 to 14:00. Table 3 shows the correlation coefficients of different variables with microinverter temperature in different time periods [47, 48]. Fig 4(a) shows the variation in microinverter and PV module temperature with irradiance level for Q.t12 PV modules and microinverter in the morning. Under conditions of low irradiance in morning hours, ambient temperature has the strongest correlation with microinverter temperature (Table 3). We find that the temperature difference between the microinverter temperature and the ambient temperature is very small (approximately 0.40°C) when irradiance is below 60 *W*/*m* ^2^. When the irradiance is greater than 60 *W*/*m* ^2^, the PV modules are heating more dramatically in addition to producing more power. Consequently, the microinverters’ temperature also starts to increase. These results are summarized in Table 4 where ΔModule.T and ΔMicro.T stand for temperature differences between the PV module temperature and the ambient temperature, and the microinverter temperature and the ambient temperature respectively.

**Table 3:** Correlation Coefficients of different variables with microinverter temperature in different time of the day.

	Morning Hours	Noontime
Irradiance	0.26	0.56
Ambient.T	0.98	0.83
Wind speed	0.05	0.07
Module.T	0.89	0.89
Power	0.13	0.53

**Table 4:** Change in PV module and microinverter temperature with irradiance during morning hours.

Irradiance	Power		ΔModule.T		ΔMicro.T	
(*W*/*m* ^2^)	(W)	SE (W)	(°C)	SE (°C)	(°C)	SE (°C)
0–60	5.64	0.18	-1.16	0.022	-0.40	0.009
> 60	61.41	1.44	4.88	0.178	1.02	0.046

At the local solar noontime, ambient temperature and PV module temperature are in strong correlation with the microinverter temperature. Irradiance, and the AC power also have a similar moderate correlation with the microinverter temperature. Temperature variance in PV modules is higher when irradiance is above 300 *W*/*m* ^2^, and PV modules temperature shows more variance than microinverter temperature (Fig 4(b)). The variance in the microinverters’ temperature due to the PV module brand variation is higher during noontime (Fig 3) since the PV module temperature rise and power output are different for different brands. The noontime summary statistics are shown in Table 5 which includes average power output, average PV module temperature and average microinverter temperature variation within different brands of PV modules.

**Table 5:** Average AC power, PV module temperature difference with ambient temperature, and microinverter temperature difference with ambient temperature at noontime.

Brand	Power		ΔModule.T		ΔMicro.T	
	W	SE(W)	°C	SE (°C)	°C	SE (°C)
P.t12	131.29	1.57	16.97	0.21	7.91	0.09
O.t12	139.45	1.64	16.50	0.20	6.97	0.07
Q.t12	124.99	1.52	17.53	0.21	7.03	0.07
R.t14	138.09	1.66	14.92	0.19	6.44	0.07
S.t14	137.70	1.68	15.80	0.20	5.84	0.07
T.t14	141.09	1.73	16.59	0.21	7.06	0.08
K.t6	140.96	1.67	15.90	0.19	6.99	0.08
L.t6	127.81	1.47	17.02	0.20	6.84	0.07

### Modeling

In pursuit of a statistical model of microinverter temperature given environmental conditions, we focus on the noontime data as the PV system is most stable and active, and and build a multiple regression model. The responsible variable is the microinverter temperature (Micro.T). The covariates include:

1. PVmodule backsheet temperature (Module.T),
2. AC power output (Power),
3. Irradiance (Irradiance), and
4. Ambient temperature (Ambient.T).

During ±2 hours around solar noontime Micro.T is highly correlated to Module.T and Ambient.T (*correlation* = 0.89 and 0.83) and moderately correlated to Irradiance and Power (*correlation* = 0.56 and 0.53). Three of the covariates, Module.T, Irradiance and Power also show high pairwise correlations among themselves (*correlations* > 0.78) among themselves, suggesting potential redundancy. Indeed, since microinverters are installed in the shadow of PV modules and are not directly exposed to sunlight (Fig 1), the effect of irradiance on Micro.T is likely through other variables. Using highly correlated covariates in the regression model can lead to unstable coefficient estimates and cryptic interpretations (collinearity) [50, 51]. One way to examine individual effects of covariates on Micro.T while holding other variables constant is through examination of the partial correlations [51] which are shown in Table 6

**Table 6:** Partial correlations between microinverter temperature and covariates: ambient temperature, PV module temperature, irradiance and AC power.

	Ambient.T	Module.T	Irradiance	Power
Partial correlation	0.727	0.674	0.008	0.006

It is clear that when holding Ambient.T and Module.T constant, the Irradiance and Power have low partial correlation with Micro.T. This is likely due to the fact that the microinverters sit behind the PV modules and do not directly exposed to sun light, and the efficiency of power conversion is high. Therefore changes in power generation do not induce large changes in microinverters.

After identifying the main contributors, we retain the dominant covariates Ambient.T and Module.T in the regression model. The interaction of Ambient.T and Module.T is also included, as well as Brand of PV modules to test if different PV brands have significant effects on the relationships. After step-wise selection and model validation, the final model is given by the following equation:

$$\begin{array}{cc} Micro.T_{i} & =\beta_{0}+\;\sum_{j=1}^{7}\beta_{0j}x_{ij}+\left(\beta_{1}+\sum_{j=1}^{7}\beta_{1j}x_{ij}\right)Ambient.T_{i}\\ & +\;\left(\beta_{2}+\sum_{j=1}^{7}\beta_{2j}x_{ij}\right)Module.T_{i}\\ & +\left(\beta_{3}+\sum_{j=1}^{7}\beta_{3j}x_{ij}\right)Ambient.T_{i}\times Module.T_{i}+\varepsilon_{i}. \end{array}$$ (1)

In Eq (1), *x*_*ij*_, *j* = 1, …, 7, *i* = 1, …, *n* are dummy variables from the first 7 brands with sum contrast. That is, *x*_*ij*_ = 1 if the i-th observation is from brand j, *x*_*ij*_ = −1 if the i-th observation is from brand 8, and *x*_*ij*_ = 0 otherwise. *ɛ*_*i*_ represents the errors associated with the predictive model for each brands of PV modules. *β*_0_, *β*_1_ and *β*_2_ are the coefficients for intercepts, Ambient.T and Module.T respectively for the mean model. The coefficient of the interactions between the Ambient.T and the Module.T in the mean model is represented by *β*_3_. Their estimations are all significant and the values are listed in Table 7.

**Table 7:** Coefficient values of different variables for mean regression predictive model for microinverter temperature.

Intercept	Ambient.T	Module.T	Ambient.T×Module.T
*β*_0_(°*C*)	*β*_1_(°*C*)	*β*_2_(°*C*)	*β*_3_ × 10^−3^(°*C* ^2^)
-1.594	0.764	0.406	-2.297

The coefficients *β*_*kj*_, *k* = 0, 1, 2, 3 depict the deviation in coefficients of intercepts, Ambient.T, the Module.T and their interaction respectively from the mean model due to the brand variation of the PV modules. For the microinverters connected to the 8th PV module brand (T.t14), the coefficients of the predictive model can be calculated by $\beta_{k8}=-(\sum_{j=1}^{7}\beta_{kj})$. The estimated values are given in Table 8

**Table 8:** List of coefficients for different variables in the predictive model due to the brand variations of the PV module.

Brands	Intercept	Ambient.T	Module.T	Ambient.T×Module.T
	*β*_0*j*_(°*C*)	*β*_1*j*_(°*C*)	*β*_2*j*_(°*C*)	*β*_3*j*_ × 10^−3^(°*C* ^2^)
S.t14	-1.494	0.094	-0.024	-0.61
R.t14	-0.440	-0.022	0.001	0.12
L.t6	0.472	-0.025	-0.016	0.50
K.t6	-0.353	-0.012	0.055	-1.34
P.t12	-0.265	-0.016	0.053	-0.71
O.t12	0.429	-0.014	-0.004	1.19
Q.t12	0.624	-0.013	-0.042	-0.73
T.t14	1.027	0.008	-0.023	1.58

The adjusted *R* ^2^ value of the regression model is 0.97 with residual standard error of 0.86°C. Furthermore the residuals of the final model do not show specific nontrivial patterns when plotted against fitted values and covariates. See discussion for a note on time related covariance structure. A QQ-plot suggests approximate normality of the errors [52]. We note that if morning and afternoon data are included in the model (fittings not shown) then both linearity and normality assumptions are not satisfied.

**Fig 5:**
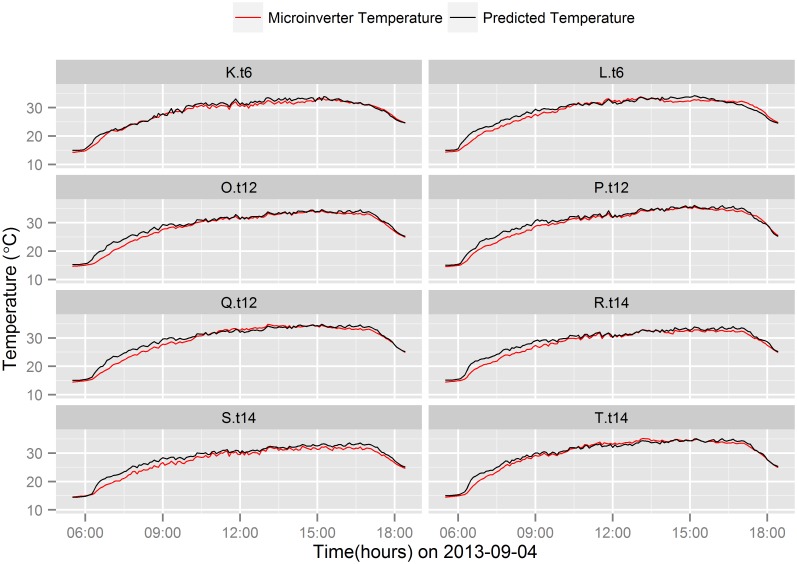
Microinverter temperature prediction comparison on a sunny day. Comparison of actual and predicted microinverter temperature on a particular sunny day (2013-09-04).

**Fig 6:**
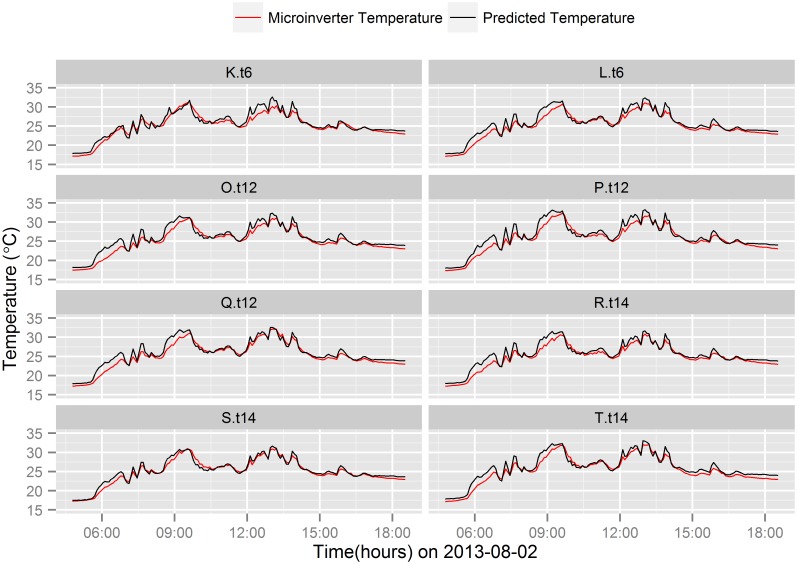
Microinverter temperature prediction comparison on a cloudy day. Comparison of actual and predicted microinverter temperature on a particular cloudy day (2013-08-02).

Figs 5 and 6 show the comparison between actual and predicted Micro.T of the microinverters connected to the 8 different brands of PV module during ±2 hours around solar noontime on a sunny day (2013-09-04) and on a cloudy day (2013-08-02), respectively. The predictive regression model predicts the Micro.T fairly well on a sunny day noontime (Fig 5), however, temperature differences between the actual and predicted Micro.T are observed during cloudy days noontime.

## Discussion

The data analytics focused on data from ±2 around solar noontime on each day when the PV system is most stable and active. Dates were identified where PV modules or the trackers were malfunctioning, and these data were removed before model fitting. In the regression predictive model, a continuous response variable (Micro.T) is predicted by both categorical (brands) and continuous (Ambient.T and the Module.T) predictors. As there is no reference brand, a sum contrast on the brands variable is used, so that the thermal performance of microinverters connected to different brands of PV modules are easily compared. In the analysis we identified that although AC power and irradiance level show moderately high correlation to microinverter temperature, the relation is mostly indirect, as shown by their small partial correlations to Micro.T with the presence of Ambient.T and the Module.T.

Irradiance is the source of energy for a PV system. However, the Micro.T is not strongly correlated with irradiance directly (Fig 2 and Table 3) since they are shaded by the PV module. Therefore, irradiance is more strongly correlated with the Module.T as compared to the Micro.T.

Figs 5 and 6 show the prediction comparison between different PV modules brands on a sunny and cloudy day, respectively. During cloudy days, the change in irradiance due to cloud cover is very sharp. The corresponding response of Module.T is very dynamic due to the direct interaction between PV module and irradiance. Therefore, the predictive model estimates rapid change in Micro.T based on the rapid changes in PV module temperature. However, the temperature rise in the microinverter is relatively slower than the predicted value due to thermal diffusivity of the microinverter. As a result, large over-prediction of Micro.T is observed in a cloudy noon (Fig 6) compared to sunny day noontime (Fig 5).

Wind speed determines the convective cooling rates of the PV module and microinverter. Surprisingly, the pair plots and correlation coefficients values from Fig 2 and Table 3, show that the Module.T and Micro.T have very little correlation with the range of wind speeds observed in SDLE SunFarm. A possible explanation of this lack of expected response is that during times of high irradiance, sustained winds were not present to cool the devices sufficiently on the minute time scale and overcome the effects of the more dominant thermal predictors, such as the radiating module. The very low correlation coefficients between wind speed and any other variable, including Module.T, strongly suggest that even at higher speeds the effect of wind on temperature is negligible. Further, at low irradiances where high wind conditions may exist, for example during storms, any change in temperature from the winds is less noticeable since the Micro.T is dominated by the Ambient.T.

Table 4 summarizes the thermal behavior of both the microinverters and the PV modules during morning hours. When the incident solar irradiance is less than 60 *W*/*m* ^2^, the Module.T and the Micro.T can actually cool down below the Ambient.T (Fig 4(a)). The PV module emissivity is greater than the ambient emissivity [53]. Therefore the PV module losses more energy through radiation during low irradiance morning hours (when irradiance < 60 *W*/*m* ^2^) than the Ambient.T, and this energy loss is not affected by PV module mounting or operational conditions [53]. In the irradiance level below 60 *W*/*m* ^2^, the AC power output of the system is very low. Therefore, energy loss during DC to AC conversion is also very small. Furthermore, the microinverters radiate more energy than it receive from the PV modules as the Module.T is lower than Micro.T during low irradiance morning hour. As a result, Micro.T is slightly below the Ambient.T.

Fig 3 shows that the temperature variation throughout the day in the microinverters connected to different brands of PV modules. The variation in thermal behavior across different PV module brands starts to show itself only during high irradiance conditions. Table 5 shows the variation of thermal behavior of Module.T and Micro.T during high irradiance local solar noontime. The largest Micro.T is observed in the microinverters connected to the P.t12 PV modules. The O.t12 PV modules provide more power than P.t12 PV modules and the P.t12 Module.T are slightly greater (approximately 0.5°C) than O.t12 Module.T. However, the P.t12 Micro.T is approximately 1°C higher than O.t12 Micro.T. This temperature difference could occur due to the backsheet emissivity difference between the P.t12 and O.t12 PV modules. The Q.t12 PV module produce approximately 16 *W* less than the T.t14 PV modules and the Q.t12 PV Module.T is, on average, 1°C is higher than the T.t14 PV module (Table 5). However, the microinverters connected to the T.t14 PV modules shows similar temperature rise to the microinverters connected to the Q.t12 microinverters due to higher Q.t12 Module.T. Similar behavior was observed among K.t6 and L.t6 Module.T and Micro.T. variables as well. These results illustrate how significant the Module.T’s influence on the Micro.T compared to the efficiency and direct internal power losses. Enphase recommends a maximum input power of 270 W, of which only 225 W will be converted by the inverter, forcing the module to operate off of maximum power, and building more heat into the module. Therefore, it may be more beneficial for the long term performance of the microinverter to power match the PV module even if the conversion efficiency is reduced, since our analysis suggests the inverter will operate at lower temperature.

Fixed rack tilted PV systems are the most common PV systems in practice. The major difference between fixed rack tilted and dual-axis tracking PV systems is: the dual-axis tracking system are always following the sun, therefore, it receives the maximum available irradiance all the time where the fixed rack system receives the maximum irradiance during solar noon. As a result, temperature of the PV modules and microinverters in dual-axis trackers rise sharply in the morning hours, reach saturation/steady state and stay in that state till evening. Temperature of the PV modules and microinverters in fixed rack tilted PV system rise slowly and reach at the saturation state only at solar noon and decrease afterwards. Fixed rack tilted PV systems are usually mounted either on open field or on rooftop. In both cases, the PV modules and microinverters receive radiation heat energy from the ground or rooftop to which the system is mount. The radiation heat energy from the ground for PV systems in dual-axis trackers is negligible due to the greater distance between ground and tracker platform. Therefore, the temperature of PV modules and microinverters in dual-axis trackers will be lower than the temperature of PV modules and microinverters in fixed rack PV systems.

## Conclusion

A multiple linear regression model for the Micro.T has been developed for the microinverters connected to different brands of PV modules installed on dual-axis trackers, which reveals the relations between microinverter temperature and most related stressors, namely Ambient.T and Module.T. A data science approach has been followed to rank-order these predictors. A further parametric model can be expanded to predict the Micro.T in fixed-rack system and roof top systems that will allow us to compare the thermal characteristics of microinverters in different PV module mountings. From the analysis, we uncover that at high irradiance, the Module.T, which is determined by the difference between absorbed solar power and maximum DC electrical power loading the PV module, is the dominant predictor of Micro.T. Our work strongly suggests that the lifetime of microinverter can be enhanced by matching power handling capability of PV module and microinverter to avoid the power saturation state of microinverter. This study summarizes the thermal behavior of microinverters on dual-axis trackers, which can be considered as baseline behavior. As time progresses, microinverters will degrade and it will effect the thermal response of the microinverters. Future work will explore the degradation of microinverters and the coupling of the data-driven thermal model with a physical model of the microinverter components to completely understand component-level degradation and their respective influence on microinverter performance, lifetime and degradation.

## Supporting Information

S1 Data Datasets for the study of microinverter thermal performance in the real-world: measurements and modeling. The comma-separated values (CSV) file contains full datasets used for this study.
(CSV)
